# Supplementary material for: Walking along chromosomes with super-resolution imaging, contact maps, and integrative modeling
Source: PLoS Genet. 2018 Dec 26;14(12):e1007872. doi: 10.1371/journal.pgen.1007872 (PMC6324821; doi:10.1371/journal.pgen.1007872)
Supplement: S1 Text — (DOCX) [file pgen.1007872.s001.docx]

**Text S1. Integrative Modeling Procedure**

To trace the chromatin structure of the imaged loci, we implemented a fitting procedure that integrates super resolution data and Hi-C interaction maps. The starting point of the procedure is an OligoSTORM density map and an ensemble of 3D models generated based on the Hi-C PGP1f mega map using TADbit [58, 59]. The approach proceeds in two main stages (Fig. S9A):

*Step 1: Rigid Body Fitting.*

In this step, we search for the optimal position and orientation of each chromosomal segment 3D model in the ensemble in the respective OligoSTORM density map (Methods) using a six-dimensional search (three translational and three rotational degrees of freedom). During the rigid body fitting procedure, we evaluate the cross-correlation coefficient (CCC, Methods) between a simulated density map from the 3D model and the OligoSTORM density map. Two keys aspects will mainly influence this first step: (i) the number of distinct high-density feature points of the map and (ii) a good initial placement of the model in an OligoSTORM density map. The initial components are manually placed in the density map, and then, for each 3D model, 100 perturbations are performed and locally optimized within the density map by steepest ascent method (Methods). Using the rigid body fitting step, we can identify the approximate position and orientation of the chromosomal segment 3D model in the density map as well as discriminate mirror models as a consequence of modeling Hi-C data in distance space rather than Cartesian space.

The goodness-of-fit in this step is assessed with a combined score (*𝐶𝑜𝑚𝑏𝑆𝑐𝑜𝑟𝑒)* of CCC with the density map and connectivity with the neighboring chromosomal segment (Methods and Fig. S11A, B).

The top-percentile best-fitting models for each chromosomal segment OligoSTORM density map were selected for the second step of the integrative protocol (with the exception of CS5 in homolog 2, which was manually fitted due to a poor connectivity score with neighboring chromosomal segments).

*Step2: Flexible Fitting Refinement*

In step 2, the conformation of the chromosomal segment was optimized simultaneously with its position and orientation in the OligoSTORM density map via flexible fitting refinement [53]. Each model in the set of best-fitting models for each chromosomal segment was subdivided into smaller rigid bodies, in agreement with the manually curated regions identified by TADbit from the Hi-C experiment as contact domains (see Methods). Then, we optimized the 3D model by refining positions and orientations of its rigid bodies with a simulated annealing rigid-body molecular dynamics protocol for up to 50 cycles. The scoring function used for the dynamic simulation in this second step included the *E*^CCC^ term with the OligoSTORM density map and terms for the satisfaction of the spatial restraints (imposed as harmonic restraints) as calculated from the Hi-C experiment. The goodness-of-fit was assessed with the CCC and the clash (CLS) scores (Table S8). We chose these scores as it has been previously shown for proteins that the improvement in CCC can be associated with the goodness-of-fit of the final models [53] and that the CLS scores are a good proxy for quantifying unfavorable steric overlaps. Generally, this stage of refinement is considered “finished” when the molecular dynamics simulation has converged as evaluated by the goodness-of-fit measures. The absence of such convergence could be related, for example, to a sub-optimal starting rigid-body conformation, an incorrect rigid-body assignment, and/or few high-density feature points in the map.
