# Supplementary material for: Walking along chromosomes with super-resolution imaging, contact maps, and integrative modeling
Source: PLoS Genet. 2018 Dec 26;14(12):e1007872. doi: 10.1371/journal.pgen.1007872 (PMC6324821; doi:10.1371/journal.pgen.1007872)
Supplement: S2 Text — (DOCX) [file pgen.1007872.s002.docx]

**Text S2. Assessment of *in-situ* Hi-C data reproducibility**

In order to assess reproducibility of our *in situ* Hi-C replicates, we calculated the Pearson’s r between Hi-C maps as a function of distance, as described in [8]. Our data remained highly correlated at all resolutions tested (see Fig. S15B for correlation as a function of the distance). To do this, we utilized a MATLAB script, which can be found here <https://www.dropbox.com/s/f8qezt6ny5v6yml/plt_correlation_distance.py?dl=0>
