# Supplementary material for: Walking along chromosomes with super-resolution imaging, contact maps, and integrative modeling
Source: PLoS Genet. 2018 Dec 26;14(12):e1007872. doi: 10.1371/journal.pgen.1007872 (PMC6324821; doi:10.1371/journal.pgen.1007872)
Supplement: S1 Table — First 9 rounds were imaged through Mainstreet, while subsequent rounds were imaged through Backstreet. Number of frames per round of imaging were 42,500–109,200. * As in Fig 2A–2D. (DOCX) [file pgen.1007872.s003.docx]

**Table S1. Chromosomal segments imaged with OligoSTORM.**

| **Round** | **Name** | **Start (hg19)** | **End (hg19)** | **Size (kb)** | **Number of oligos** | **Average # of localizations/**  **Cluster*** |
| --- | --- | --- | --- | --- | --- | --- |
| 1 | CS1 | 7,400,000 | 8,680,000 | 1,280 | 7,861 | 4,825 |
| 2 | CS2 | 8,680,000 | 9,920,000 | 1,240 | 5,489 | 2,979 |
| 3 | CS3 | 9,920,000 | 11,720,000 | 1,800 | 10,985 | 4,489 |
| 4 | CS4 | 11,720,000 | 12,760,000 | 1,040 | 3,768 | 2,759 |
| 5 | CS5 | 12,760,000 | 13,320,000 | 560 | 4,516 | 2,560 |
| 6 | CS6 | 13,320,000 | 13,840,000 | 520 | 2,720 | 1,861 |
| 7 | CS7 | 13,840,000 | 14,680,000 | 840 | 4,601 | 2,620 |
| 8 | CS8 | 14,680,000 | 15,200,000 | 520 | 2,624 | 2,068 |
| 9 | CS9 | 15,200,000 | 15,560,000 | 360 | 2,843 | 1,389 |
| 10 | CD51 | 13,840,000 | 13,980,000 | 140 | 751 | 1,045 |
| 11 | CD52 | 13,980,000 | 14,240,000 | 260 | 1,698 | 1,526 |
| 12 | CD53 | 14,240,000 | 14,590,000 | 350 | 1,639 | 1,760 |
| 13 | CD54 | 14,590,000 | 14,680,000 | 90 | 513 | 516 |
| 14 | Loop anchor in CS 6 | 13,400,000 | 13,410,000 | 10 | 41 | 258 |
| 15 | Loop body in CS 6 | 13,410,000 | 13,680,000 | 270 | 1,473 | 3,513 |
| 16 | Loop anchor in CS 6 | 13,680,000 | 13,690,000 | 10 | 28 | 586 |
| 17 | Downstream loop flank in CS 6 | 13,690,000 | 13,710,000 | 20 | 104 | 201 |
| 18 | Upstream loop flank in CS 6 | 13,320,000 | 13,400,000 | 80 | 604 | 2,292 |
| 19 | *DNMT1* gene | 10,244,022 | 10,303,505 | 59.484 | 529 | 1,078 |
| 20 | DNMT1 DMR | 10,303,506 | 10,306,415 | 2.91 | 18 | 56 |
