# Supplementary material for: Walking along chromosomes with super-resolution imaging, contact maps, and integrative modeling
Source: PLoS Genet. 2018 Dec 26;14(12):e1007872. doi: 10.1371/journal.pgen.1007872 (PMC6324821; doi:10.1371/journal.pgen.1007872)
Supplement: S2 Table — (A) Walk along chromosome 5. (B) Walk along chromosome 3. * As in Fig 2E. (DOCX) [file pgen.1007872.s004.docx]

**Table S2. Multiple chromosome walk.**

A.

| **Round** | **Start (hg19)** | **End (hg19)** | **Size (kb)** | **Number of oligos** | **Average # of localizations/cluster*** |
| --- | --- | --- | --- | --- | --- |
| 5 | 120,000,000 | 120,250,000 | 250 | 864 | 558 |
| 6 | 120,250,000 | 120,500,000 | 250 | 989 | 652 |
| 7 | 120,500,000 | 120,750,000 | 250 | 684 | 513 |
| 8 | 120,750,000 | 121,000,000 | 250 | 547 | 198 |

B.

| **Round** | **Start (hg19)** | **End (hg19)** | **Size (kb)** | **Number of oligos** | **Average # of localizations/cluster*** |
| --- | --- | --- | --- | --- | --- |
| 6 | 150,000,000 | 150,500,000 | 500 | 2,524 | 1,503 |
| 7 | 150,500,000 | 151,000,000 | 500 | 3,467 | 2,320 |
| 8 | 151,000,000 | 151,500,000 | 500 | 2,511 | 878 |
