# Supplementary material for: Walking along chromosomes with super-resolution imaging, contact maps, and integrative modeling
Source: PLoS Genet. 2018 Dec 26;14(12):e1007872. doi: 10.1371/journal.pgen.1007872 (PMC6324821; doi:10.1371/journal.pgen.1007872)
Supplement: S3 Table — * As in Fig 2F. Number of frames for each segment was 30,000. (DOCX) [file pgen.1007872.s005.docx]

**Table S3. Chromosomal segments imaged with OligoDNA-PAINT**.

| **Segment** | **Imager** | **Imager conc. (nM)** | **# of localizations*** | **Median fit precision (nm)** | **NeNA localization precision in XY**  **(nm)** | **Supported resolution in XY**  **(nm)** | **On (s)** | **Off (s)** |
| --- | --- | --- | --- | --- | --- | --- | --- | --- |
| CS7 | P1 | 1 | 5,462 | 4.30 | 9.43 | 22.15 | 3.11 | 10.85 |
| CS8 | P13 | 1 | 6,973 | 2.96 | 8.00 | 18.79 | 2.64 | 8.75 |
| CS9 | P9 | 1 | 11,348 | 2.33 | 5.40 | 12.68 | 3.45 | 7.85 |
