# Supplementary material for: Walking along chromosomes with super-resolution imaging, contact maps, and integrative modeling
Source: PLoS Genet. 2018 Dec 26;14(12):e1007872. doi: 10.1371/journal.pgen.1007872 (PMC6324821; doi:10.1371/journal.pgen.1007872)
Supplement: S4 Table — Chromosomal segment (CS); Active, % active CS; Inactive, % inactive CS; Imaging classification; Hi-C classification (Methods). (DOCX) [file pgen.1007872.s006.docx]

**Table S4. Compartment classification.**

| CS | Active | Inactive | Imaging classification | Hi-C classification |
| --- | --- | --- | --- | --- |
| 4 | 87 | 13 | A | A |
| 3 | 84 | 16 | A | A |
| 5 | 84 | 16 | A | A |
| 7 | 82 | 18 | A | A |
| 6 | 79 | 21 | A | A |
| 1 | 58 | 42 | A | A |
| 9 | 37 | 63 | B | A |
| 2 | 26 | 74 | B | B |
| 8 | 24 | 76 | B | B |
