# Supplementary material for: Walking along chromosomes with super-resolution imaging, contact maps, and integrative modeling
Source: PLoS Genet. 2018 Dec 26;14(12):e1007872. doi: 10.1371/journal.pgen.1007872 (PMC6324821; doi:10.1371/journal.pgen.1007872)
Supplement: S5 Table — (DOCX) [file pgen.1007872.s007.docx]

**Table S5. Hi-C library statistics for PGP1f “mega map” (combined data from ten libraries).**

|  | **Combined data from 10 libraries** |
| --- | --- |
| **Sequenced Read Pairs (Status)** | 2,599,264,644 |
| **Normal Paired** | 1,327,845,816 (51.09%) |
| **Chimeric Paired** | 1,054,718,010 (40.58%) |
| **Chimeric Ambiguous** | 192,254,720 (7.40%) |
| **Unalignable** | 24,446,098 (0.94%) |
| **Alignable** | 2,382,563,826 (91.66%) |
| **Unique Read Pairs** | 2,177,858,724 |
| **Optical Duplicates** | 194,556,665 |
| **Intra-fragment Read Pairs** | 10,148,437 |
| **Below MAPQ Threshold** | 17,681,295 (0.68% / 0.81%) |
| **Hi-C Contacts** | 197,791,801 (7.61% / 9.08%) |
| **Ligation Motif Present** | 1,962,385,628 (75.50% / 90.11%) |
| **3' Bias (Long Range)** | 808,781,294 (31.12% / 37.14%) |
| **Pair Type % (L-I-O-R)** | 75% - 25% |
| **Inter-chromosomal** | 25% - 25% - 25% - 25% |
| **Intra-chromosomal** | 606,574,526 (23.34% / 27.85%) |
| **Short Range (<20Kb)** | 1,355,811,102 (52.16% / 62.25%) |
| **Long Range (>20Kb)** | 320,750,057 (12.34% / 14.73%) |
