# Supplementary material for: Walking along chromosomes with super-resolution imaging, contact maps, and integrative modeling
Source: PLoS Genet. 2018 Dec 26;14(12):e1007872. doi: 10.1371/journal.pgen.1007872 (PMC6324821; doi:10.1371/journal.pgen.1007872)
Supplement: S6 Table — Nucleus ID; ES1, ellipticity score for the first homolog; ES2, ellipticity score for the second homolog; ER, ellipticity ratio. (DOCX) [file pgen.1007872.s008.docx]

**Table S6. Homologs Ellipticity** **in the cell population.**

| Nucleus | ES1 | ES2 | ER |
| --- | --- | --- | --- |
| 1 | 2.58 | 6.73 | 2.61 |
| 2 | 1.87 | 1.56 | 1.20 |
| 3 | 1.22 | 2.07 | 1.70 |
| 4 | 1.82 | 2.80 | 1.54 |
| 5 | 1.56 | 2.26 | 1.45 |
| 6 | 12.00 | 4.17 | 2.88 |
| 7 | 2.29 | 2.72 | 1.19 |
| 8 | 2.36 | 6.52 | 2.77 |
| 9 | 1.70 | 3.80 | 2.23 |
| 10 | 3.03 | 1.23 | 2.46 |
| 11 | 8.79 | 2.47 | 3.56 |
| 12 | 1.88 | 6.11 | 3.24 |
| 13 | 1.88 | 4.45 | 2.37 |
| 14 | 1.60 | 2.92 | 1.82 |
| 15 | 2.27 | 5.82 | 2.56 |
| 16 | 1.87 | 2.74 | 1.46 |
| 17 | 4.38 | 2.57 | 1.70 |
| 18 | 5.40 | 1.90 | 2.84 |
