# Supplementary material for: Walking along chromosomes with super-resolution imaging, contact maps, and integrative modeling
Source: PLoS Genet. 2018 Dec 26;14(12):e1007872. doi: 10.1371/journal.pgen.1007872 (PMC6324821; doi:10.1371/journal.pgen.1007872)
Supplement: S7 Table — Chromosomal segment (CS); Start and End, genomic location; MMPscore, the matrix modeling potential score; pSCC, predicted distance Spearman Cross Correlation for the predicted accuracy of the models; SCC, Spearman Cross Correlation between Hi-C contact frequencies and the model-based contact map. (DOCX) [file pgen.1007872.s009.docx]

**Table S7. Assessment of potential for modeling of the analyzed regions.**

| CS | Start | End | MMPscore | pSCC |  | SCC |
| --- | --- | --- | --- | --- | --- | --- |
| 1 | 7,400,000 | 8,680,000 | 0.807 | 0.79 |  | 0.8731 |
| 2 | 8,680,000 | 9,920,000 | 0.792 | 0.77 |  | 0.8286 |
| 3 | 9,920,000 | 11,720,000 | 0.800 | 0.78 |  | 0.8806 |
| 4 | 11,720,000 | 12,760,000 | 0.786 | 0.763 |  | 0.8457 |
| 5 | 12,760,000 | 13,320,000 | 0.82 | 0.811 |  | 0.8875 |
| 6 | 13,320,000 | 13,840,000 | 0.794 | 0.773 |  | 0.8677 |
| 7 | 13,840,000 | 14,680,000 | 0.808 | 0.794 |  | 0.8701 |
| 8 | 14,680,000 | 15,200,000 | 0.783 | 0.757 |  | 0.8380 |
| 9 | 15,200,000 | 15,560,000 | 0.787 | 0.764 |  | 0.7670 |
| full | 7,400,000 | 15,560,000 | 0.659 | 0.58 |  | 0.8301 |
