# Supplementary material for: Walking along chromosomes with super-resolution imaging, contact maps, and integrative modeling
Source: PLoS Genet. 2018 Dec 26;14(12):e1007872. doi: 10.1371/journal.pgen.1007872 (PMC6324821; doi:10.1371/journal.pgen.1007872)
Supplement: S8 Table — Chromosomal segment (CS); Cross Correlation Coefficient of the starting rigid fitted model (CCCs); Cross Correlation Coefficient of the final model after flexible fitting refinement (CCCf); Clash Score of the starting rigid fitted model (CLSs); Clash Score of the final model after flexible fitting refinement (CLSf); Root Mean Square Deviation between starting rigid fitted model and final model after flexible fitting refinement (RMSDs-f). (DOCX) [file pgen.1007872.s010.docx]

**Table S8. Flexible fitting refinement: assessment of the goodness-of-fit.**

| CS | CCCs | | CCCf | | CLSs | | CLSf | | RMSDs-f | |
| --- | --- | --- | --- | --- | --- | --- | --- | --- | --- | --- |
| 1 | 0.47 | 0.59 | 0.61 | 0.83 | 0.17 | 0.40 | 0.20 | 0.50 | 14.32 | 19.13 |
| 2 | 0.57 | 0.53 | 0.72 | 0.76 | 0.45 | 0.26 | 0.55 | 0.30 | 16.20 | 22.23 |
| 3 | 0.50 | 0.42 | 0.74 | 0.70 | 0.60 | 0.58 | 0.66 | 0.65 | 20.90 | 22.76 |
| 4 | 0.64 | 0.60 | 0.77 | 0.71 | 0.34 | 0.36 | 0.40 | 0.45 | 19.43 | 10.35 |
| 5 | 0.62 | 0.53 | 0.69 | 0.78 | 0.14 | 0.10 | 0.16 | 0.13 | 13.90 | 19.27 |
| 6 | 0.68 | 0.60 | 0.78 | 0.81 | 0.16 | 0.16 | 0.21 | 0.17 | 16.30 | 16.30 |
| 7 | 0.66 | 0.55 | 0.82 | 0.84 | 0.25 | 0.25 | 0.32 | 0.35 | 18.89 | 17.29 |
| 8 | 0.70 | 0.63 | 0.82 | 0.82 | 0.20 | 0.19 | 0.22 | 0.24 | 15.20 | 17.42 |
| 9 | 0.78 | 0.72 | 0.85 | 0.90 | 0.12 | 0.12 | 0.17 | 0.18 | 16.00 | 14.48 |
