# Supplementary material for: Walking along chromosomes with super-resolution imaging, contact maps, and integrative modeling
Source: PLoS Genet. 2018 Dec 26;14(12):e1007872. doi: 10.1371/journal.pgen.1007872 (PMC6324821; doi:10.1371/journal.pgen.1007872)
Supplement: S9 Table — (DOCX) [file pgen.1007872.s011.docx]

**Table S9. Hi-C library statistics for PGP1f replicates.**

|  | **Library 1** | **Library 2** | **Library 3** | **Library 4** | **Library 5** | **Library 6** | **Library 7** | **Library 8** | **Library 9** | **Library 10** |
| --- | --- | --- | --- | --- | --- | --- | --- | --- | --- | --- |
| **Sequenced Read Pairs (Status)** | 299,490,370 | 392,130,190 | 260,187,521 | 240,853,363 | 353,731,237 | 244,315,522 | 276,051,774 | 182,202,553 | 148,230,293 | 202,071,821 |
| **Normal Paired** | 169,241,991 (56.51%) | 191,366,037 (48.80%) | 131,616,224 (50.59%) | 117,131,260 (48.63%) | 172,145,042 (48.67%) | 113,747,955 (46.56%) | 136,663,677 (49.51%) | 104,109,923 (57.14%) | 84,858,979 (57.25%) | 106,964,728 (52.93%) |
| **Chimeric Paired** | 102,483,082 (34.22%) | 170,744,052 (43.54%) | 107,853,468 (41.45%) | 104,434,064 (43.36%) | 151,879,044 (42.94%) | 110,385,544 (45.18%) | 117,010,889 (42.39%) | 62,813,418 (34.47%) | 50,185,335 (33.86%) | 76,929,114 (38.07%) |
| **Chimeric Ambiguous** | 19,764,751 (6.60%) | 25,286,304 (6.45%) | 19,382,175 (7.45%) | 18,280,954 (7.59%) | 27,321,747 (7.72%) | 18,757,436 (7.68%) | 20,929,819 (7.58%) | 14,038,641 (7.70%) | 12,073,840 (8.15%) | 16,419,053 (8.13%) |
| **Unalignable** | 8,000,546 (2.67%) | 4,733,797 (1.21%) | 1,335,654 (0.51%) | 1,007,085 (0.42%) | 2,385,404 (0.67%) | 1,424,587 (0.58%) | 1,447,389 (0.52%) | 1,240,571 (0.68%) | 1,112,139 (0.75%) | 1,758,926 (0.87%) |
| **Ligation Motif Present** | 172,812,075 (57.70%) | 273,415,502 (69.73%) | 157,679,461 (60.60%) | 154,700,894 (64.23%) | 209,825,881 (59.32%) | 156,587,941 (64.09%) | 166,374,539 (60.27%) | 101,327,897 (55.61%) | 80,034,644 (53.99%) | 122,759,498 (60.75%) |
| **Alignable (Normal+Chimeric Paired)** | 271,725,073 (90.73%) | 362,110,089 (92.34%) | 239,469,692 (92.04%) | 221,565,324 (91.99%) | 324,024,086 (91.60%) | 224,133,499 (91.74%) | 253,674,566 (91.89%) | 166,923,341 (91.61%) | 135,044,314 (91.10%) | 183,893,842 (91.00%) |
| **Unique Read Pairs** | 258,780,678 (86.41%) | 339,925,404 (86.69%) | 217,563,592 (83.62%) | 202,061,790 (83.89%) | 291,251,534 (82.34%) | 203,252,621 (83.19%) | 230,970,961 (83.67%) | 148,893,673 (81.72%) | 121,335,775 (81.86%) | 163,822,696 (81.07%) |
| **PCR Duplicates** | 12,074,226 (4.03%) | 20,980,766 (5.35%) | 20,786,631 (7.99%) | 18,529,507 (7.69%) | 31,251,510 (8.83%) | 19,894,510 (8.14%) | 21,570,008 (7.81%) | 17,139,552 (9.41%) | 13,048,212 (8.80%) | 19,281,743 (9.54%) |
| **Optical Duplicates** | 870,169 (0.29%) | 1,203,919 (0.31%) | 1,119,469 (0.43%) | 974,027 (0.40%) | 1,521,042 (0.43%) | 986,368 (0.40%) | 1,133,597 (0.41%) | 890,116 (0.49%) | 660,327 (0.45%) | 789,403 (0.39%) |
| **Library Complexity Estimate** | 2,947,002,205 | 2,982,605,992 | 1,285,861,304 | 1,238,446,189 | 1,554,717,822 | 1,175,923,388 | 1,392,934,261 | 747,840,539 | 646,452,946 | 807,232,888 |
| **Intra-fragment Read Pairs** | 2,212,105 (0.74% / 0.85%) | 3,528,694 (0.90% / 1.04%) | 1,131,554 (0.43% / 0.52%) | 1,412,704 (0.59% / 0.70%) | 3,750,411 (1.06% / 1.29%) | 1,226,448 (0.50% / 0.60%) | 1,386,607 (0.50% / 0.60%) | 941,268 (0.52% / 0.63%) | 996,598 (0.67% / 0.82%) | 1,094,906 (0.54% / 0.67%) |
| **Below MAPQ Threshold** | 22,946,825 (7.66% / 8.87%) | 29,492,420 (7.52% / 8.68%) | 19,200,033 (7.38% / 8.83%) | 18,069,839 (7.50% / 8.94%) | 26,825,476 (7.58% / 9.21%) | 18,578,808 (7.60% / 9.14%) | 20,532,635 (7.44% / 8.89%) | 14,192,055 (7.79% / 9.53%) | 11,776,093 (7.94% / 9.71%) | 16,177,617 (8.01% / 9.88%) |
| **Hi-C Contacts** | 233,621,748 (78.01% / 90.28%) | 306,904,290 (78.27% / 90.29%) | 197,232,005 (75.80% / 90.65%) | 182,579,247 (75.81% / 90.36%) | 260,675,647 (73.69% / 89.50%) | 183,447,365 (75.09% / 90.26%) | 209,051,719 (75.73% / 90.51%) | 133,760,350 (73.41% / 89.84%) | 108,563,084 (73.24% / 89.47%) | 146,550,173 (72.52% / 89.46%) |
| **Ligation Motif Present** | 92,975,257 (31.04% / 35.93%) | 147,078,375 (37.51% / 43.27%) | 79,083,551 (30.39% / 36.35%) | 77,797,083 (32.30% / 38.50%) | 102,795,361 (29.06% / 35.29%) | 77,819,423 (31.85% / 38.29%) | 83,386,580 (30.21% / 36.10%) | 49,349,789 (27.09% / 33.14%) | 38,907,938 (26.25% / 32.07%) | 59,587,937 (29.49% / 36.37%) |
| **3' Bias (Long Range)** | 69% - 31% | 74% - 26% | 75% - 25% | 76% - 24% | 77% - 23% | 77% - 23% | 76% - 24% | 72% - 28% | 73% - 27% | 75% - 25% |
| **Pair Type % (L-I-O-R)** | 25% - 25% - 25% - 25% | 25% - 25% - 25% - 25% | 25% - 25% - 25% - 25% | 25% - 25% - 25% - 25% | 25% - 25% - 25% - 25% | 25% - 25% - 25% - 25% | 25% - 25% - 25% - 25% | 25% - 25% - 25% - 25% | 25% - 25% - 25% - 25% | 25% - 25% - 25% - 25% |
| **Inter-chromosomal** | 55,054,421 (18.38% / 21.27%) | 74,848,460 (19.09% / 22.02%) | 48,691,205 (18.71% / 22.38%) | 46,321,395 (19.23% / 22.92%) | 75,386,236 (21.31% / 25.88%) | 48,500,985 (19.85% / 23.86%) | 50,628,708 (18.34% / 21.92%) | 65,223,802 (35.80% / 43.81%) | 60,341,635 (40.71% / 49.73%) | 81,577,679 (40.37% / 49.80%) |
| **Intra-chromosomal** | 178,567,327 (59.62% / 69.00%) | 232,055,830 (59.18% / 68.27%) | 148,540,800 (57.09% / 68.27%) | 136,257,852 (56.57% / 67.43%) | 185,289,411 (52.38% / 63.62%) | 134,946,380 (55.23% / 66.39%) | 158,423,011 (57.39% / 68.59%) | 68,536,548 (37.62% / 46.03%) | 48,221,449 (32.53% / 39.74%) | 64,972,494 (32.15% / 39.66%) |
| **Short Range (<20Kb)** | 48,073,771 (16.05% / 18.58%) | 65,627,376 (16.74% / 19.31%) | 33,253,707 (12.78% / 15.28%) | 30,740,698 (12.76% / 15.21%) | 39,045,007 (11.04% / 13.41%) | 30,392,765 (12.44% / 14.95%) | 35,222,390 (12.76% / 15.25%) | 13,659,808 (7.50% / 9.17%) | 9,888,539 (6.67% / 8.15%) | 14,845,996 (7.35% / 9.06%) |
| **Long Range (>20Kb)** | 130,493,315 (43.57% / 50.43%) | 166,428,281 (42.44% / 48.96%) | 115,286,983 (44.31% / 52.99%) | 105,517,081 (43.81% / 52.22%) | 146,244,347 (41.34% / 50.21%) | 104,553,567 (42.79% / 51.44%) | 123,200,494 (44.63% / 53.34%) | 54,876,465 (30.12% / 36.86%) | 38,332,773 (25.86% / 31.59%) | 50,126,260 (24.81% / 30.60%) |
