# Supplementary figures and images for: Walking along chromosomes with super-resolution imaging, contact maps, and integrative modeling
Source: PLoS Genet. 2018 Dec 26;14(12):e1007872. doi: 10.1371/journal.pgen.1007872 (PMC6324821; doi:10.1371/journal.pgen.1007872)

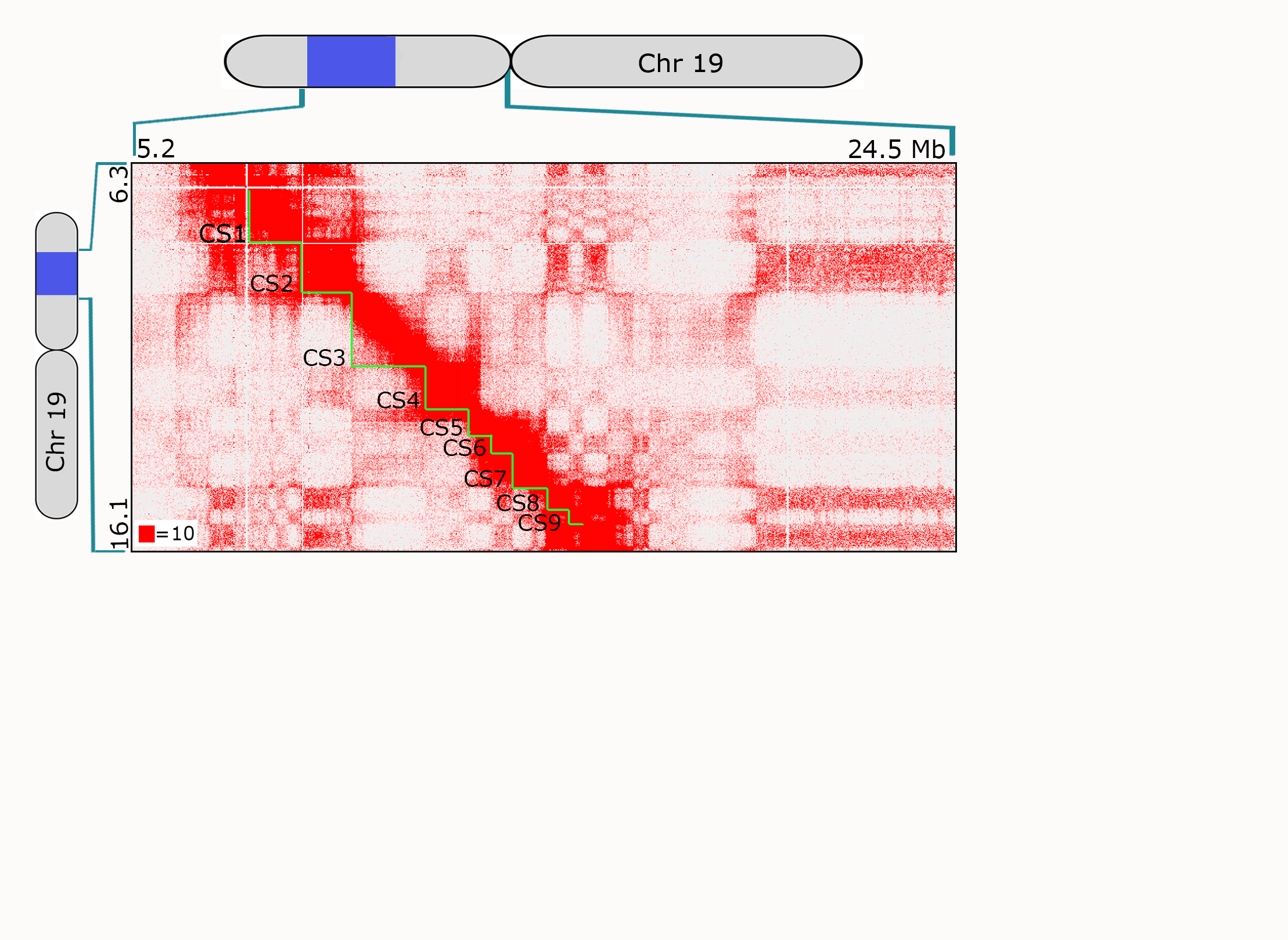

Supplement: S1 Fig — Hi-C map of region including CS1-9, displayed at 25 kb resolution. (See Methods for data processing and normalization.) Contact count indicated by the color of each pixel, ranging from 0 (white) to 10 (red); OligoSTORM steps (CS1-9) delineated in green. Cartoons of chromosome 19 show extent of the 8.16 Mb imaged region (blue). (TIF) [file pgen.1007872.s012.tif]

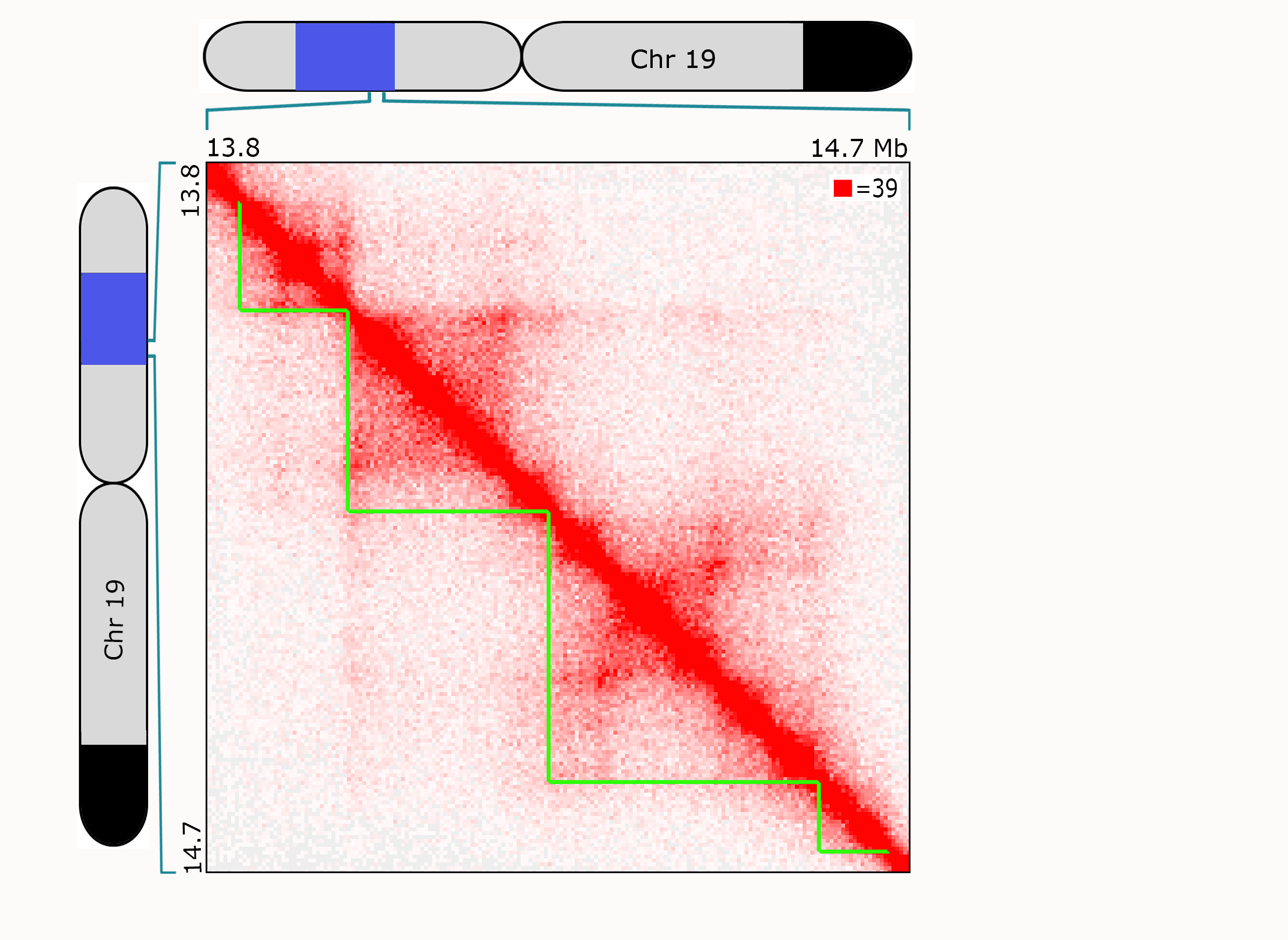

Supplement: S2 Fig — Hi-C map of region including CS7, displayed at 5 kb resolution. (See Methods for data processing and normalization.) Contact count indicated by the color of each pixel, ranging from 0 (white) to 39 (red); 4 subregions of CS7 delineated in green. Cartoons of chromosome 19 show extent of the 8.16 Mb imaged region (blue) and block of SNVs shared between PGP1 and PGP95 (black; as in Fig 1B). (TIF) [file pgen.1007872.s013.tif]

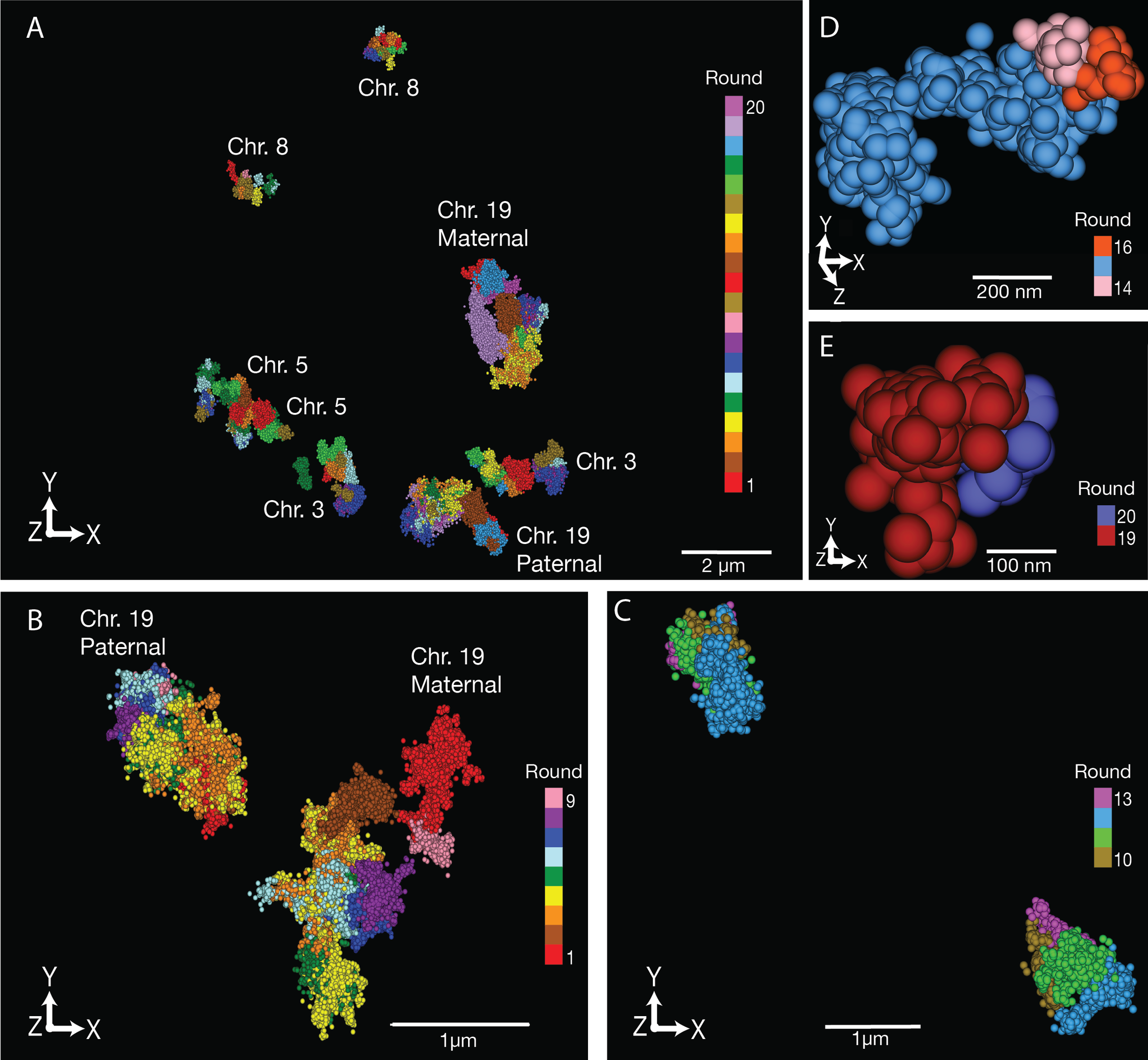

Supplement: S3 Fig — (A) Diploid nucleus showing walks along multiple chromosomes. Walks were initiated in rounds 4, 5, and 6 on Chromosomes 8, 5, and 3 in step sizes of 100, 250, and 500 kb, respectively, while simultaneously walking along Chromosome 19, commencing in round 1 and in step sizes ranging from 3 to 1,800 kb. This walk encompassed a total of 23 cycles (see color bar) and, although it was not successful in all rounds on all chromosomes, it nevertheless illustrates the potential of this approach to increase throughput. Parent-of-origin identified with HOPs, as described later in the text. (B, C) Another diploid nucleus showing 9 chromosomal segments (B, ranging in size from 0.36 to 1.8 Mb; parental origin identified with HOPs) and the four subregions comprising the 840 kb of chromosomal segment 7 (C, subregions ranging in size from 90 to 350 kb). (D) A loop (290 kb), including its anchors (pink and orange; 10 kb each) and loop body (blue; 270 kb). (E) DNMT1 gene (59.5 kb) and its DMR (2.9 kb). For more details, refer to S1 and S2 Tables. (TIF) [file pgen.1007872.s014.tif]

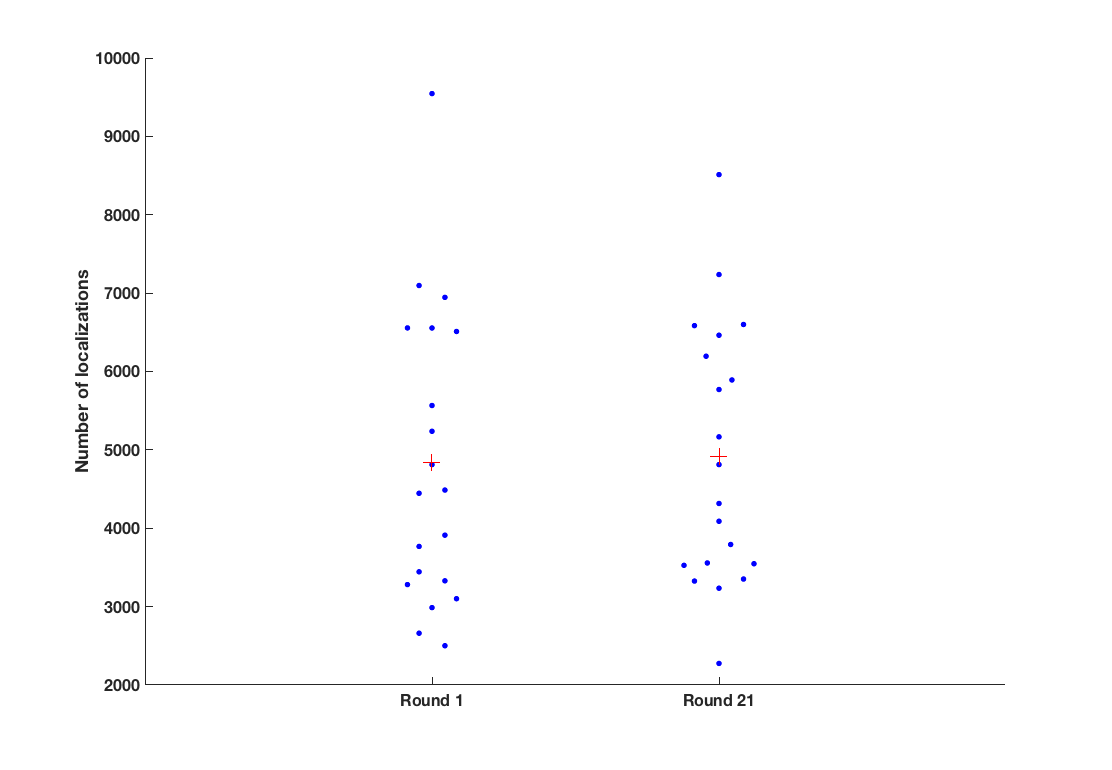

Supplement: S4 Fig — After querying the change in fractional overlap for CS1 after 21 rounds of imaging with OligoSTORM (Fig 2G), we assessed whether the number of localizations had changed using the localizations detected from 20 homologs (total of 10 nuclei, colored as blue dots; Methods). The mean number of localizations per cluster (red cross) is similar between rounds 1 and 21, implying no significant change (two-sample Kolmogorov-Smirnov test, p = 0.9655). (TIF) [file pgen.1007872.s015.tif]

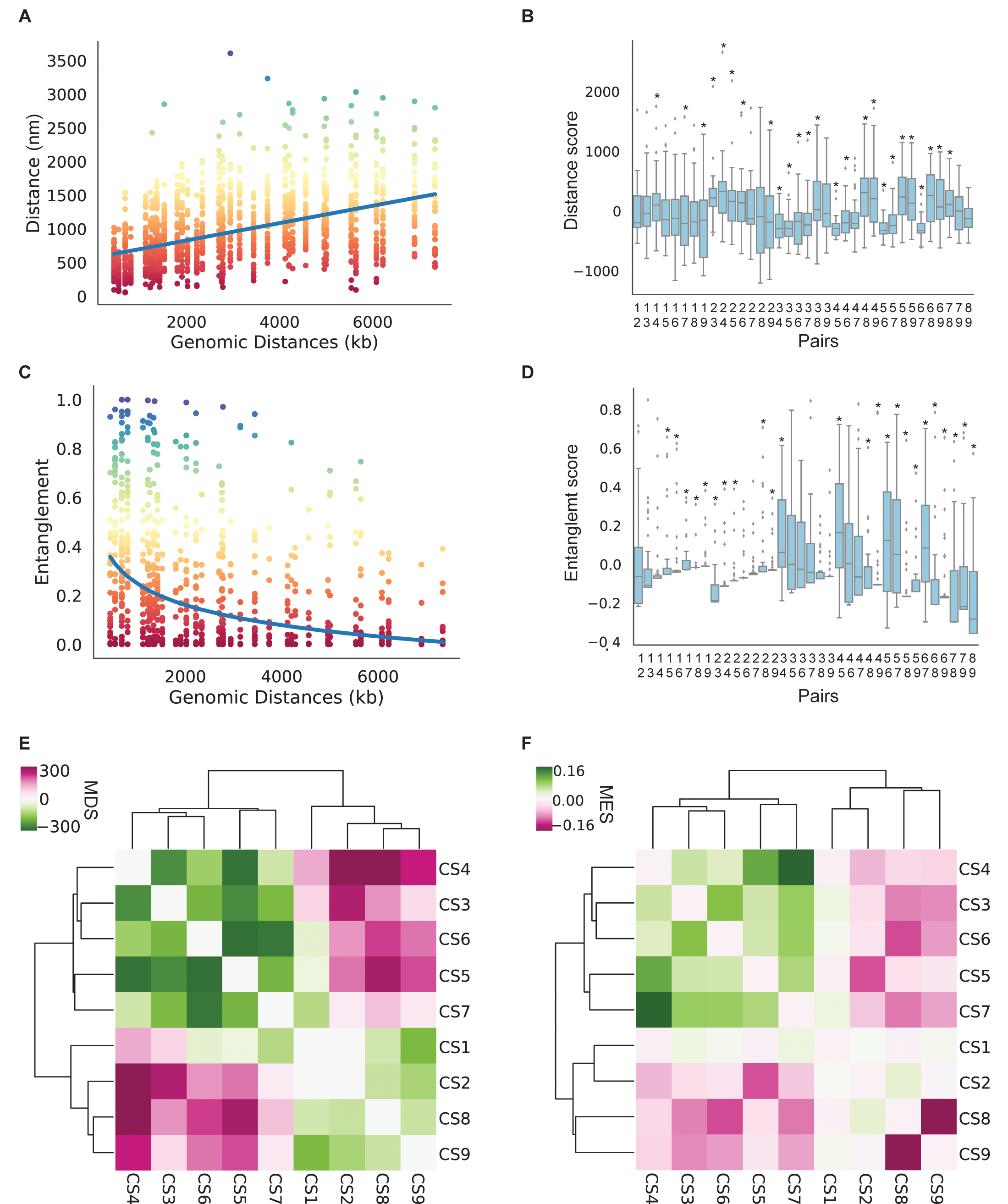

Supplement: S5 Fig — (A) Distance versus genomic distance for the 342 analyzed chromosomal segments using a power-law function fitted to the data (blue line). Data are colored by their distance value (rainbow color palette). (B) Distribution and variation of the distance score (DS) of the two chromosomal segments being compared. (C) Entanglement versus genomic distance for the 342 analyzed chromosomal segments with a power-law function fitted to the data (blue line). Data are colored by their entanglement value (rainbow color palette). (D) Distribution and variation of the entanglement score (ES) of the two chromosomal segments being compared. (B, D) Box boundaries represent 1st and 3rd quartiles, middle line represents median, and whiskers extend to 1.5 times the interquartile range, (Mann-Whitney rank, *: p < 5 x 10^−2^). (E, F) Hierarchical clustering of (E) mean distance scores (MDS) and (F) mean entanglement scores (MES) reveal two major groups of chromosomal segments. (TIF) [file pgen.1007872.s016.tif]

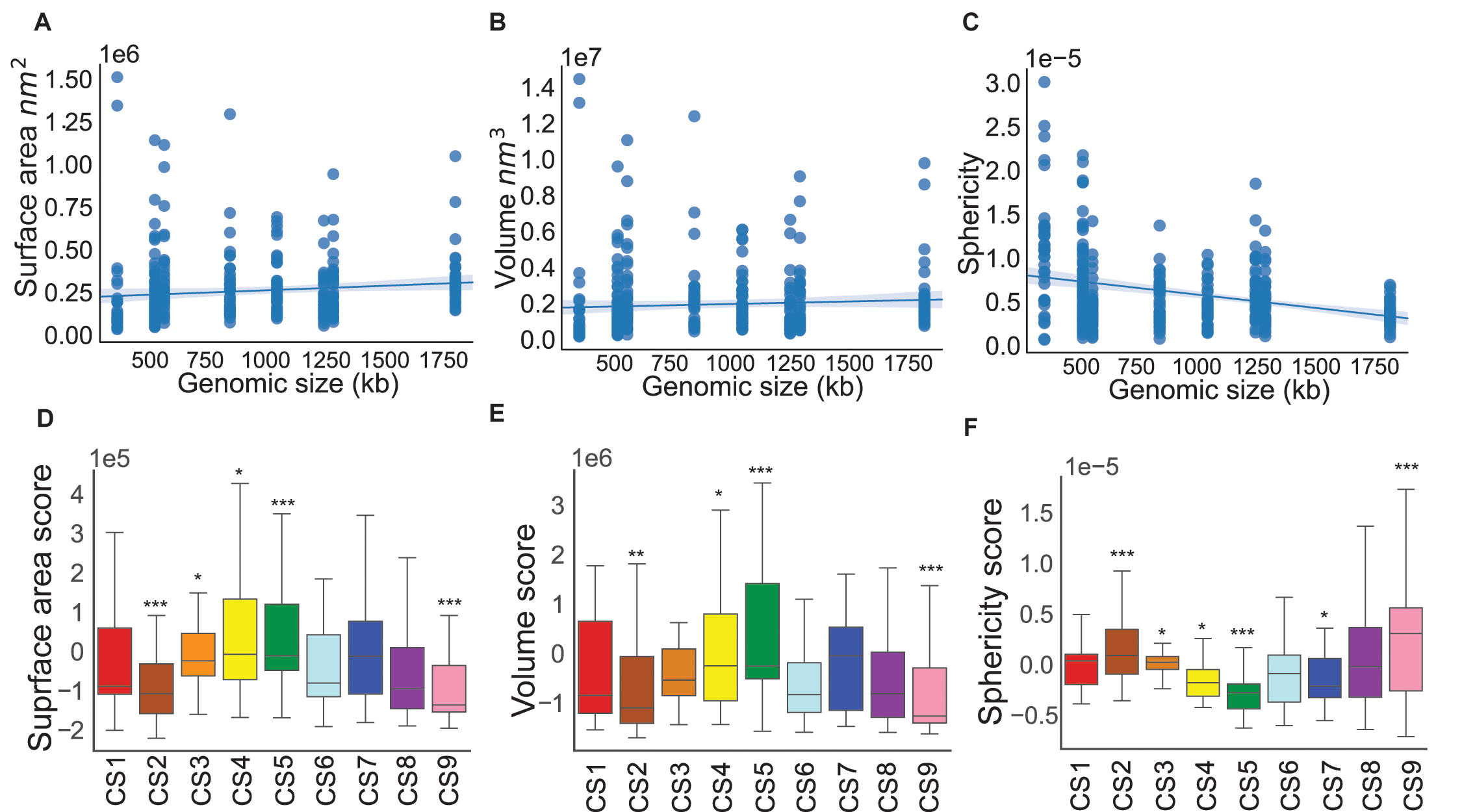

Supplement: S6 Fig — (A-C) Surface area, volume and sphericity versus genomic distance for the 342 analyzed chromosomal segments (linear fit in blue). (D-F) Box plots showing distribution and variation of the 9 chromosomal segments in terms of (D) surface area, (E) volume, and (F) sphericity score. The chromosomal segments are color-coded as in Fig 2A. Box boundaries represent 1st and 3rd quartiles, middle line represents median, and whiskers extend to 1.5 times the interquartile range (Mann-Whitney rank test, *: p < 5 x 10^−2^, **: p < 10−2, ***: p < 10−3). (TIF) [file pgen.1007872.s017.tif]

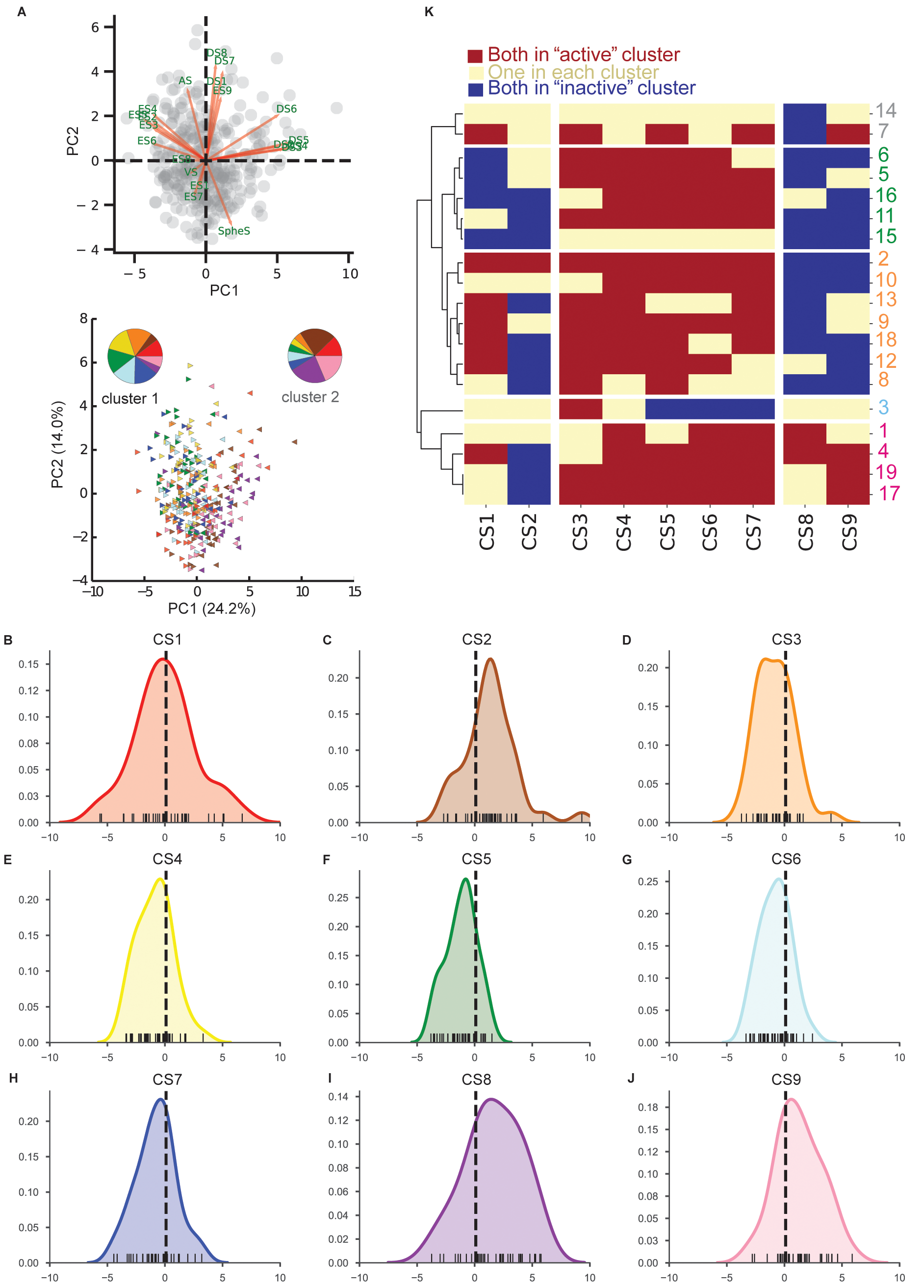

Supplement: S7 Fig — (A) Unsupervised PCA of 3D features for the 342 visualized chromosomal segments, the 3D features being: surface area, volume, sphericity, entanglement, and distance scores. Upper panel, PCA biplot (combination of score plot and loading plot) assessing the relationships between the loadings and the variables of the first two components. The lengths of the arrows indicate how each original variable contributes to the component (variables that have little contribution have almost zero length). The direction of the vectors indicates how two variables are correlated. Aligned vectors are strongly positively correlated between each other, opposite vectors indicate negative correlation, and vectors at right angles indicate no correlation. Each data point coloured in grey represents a chromosomal segment. Lower panel, PCA of 3D features for the 342 visualized chromosomal segments, each chromosomal segment colored as in Fig 2A and 2B, with relative abundances displayed as pie charts. (B-J) Distribution along PC1 for CS1-9, color coded as in Fig 2. Vertical black dotted line represents the partition of cluster 1 and cluster 2. (K) Hierarchical clustering of the compartment state profile vectors for the 19 analyzed nuclei results in 5 large clusters. The compartment state profile (Methods) was calculated comparing the chromatin state profile of each homolog within a single nucleus. Note, the two most populated clusters confirm that, in the majority of the analyzed nuclei, the compartment state profile is maintained with CS2, 8, and 9 mostly inactive, CS3-7 mostly active, and CS1seeming to occupy both states. (TIF) [file pgen.1007872.s018.tif]

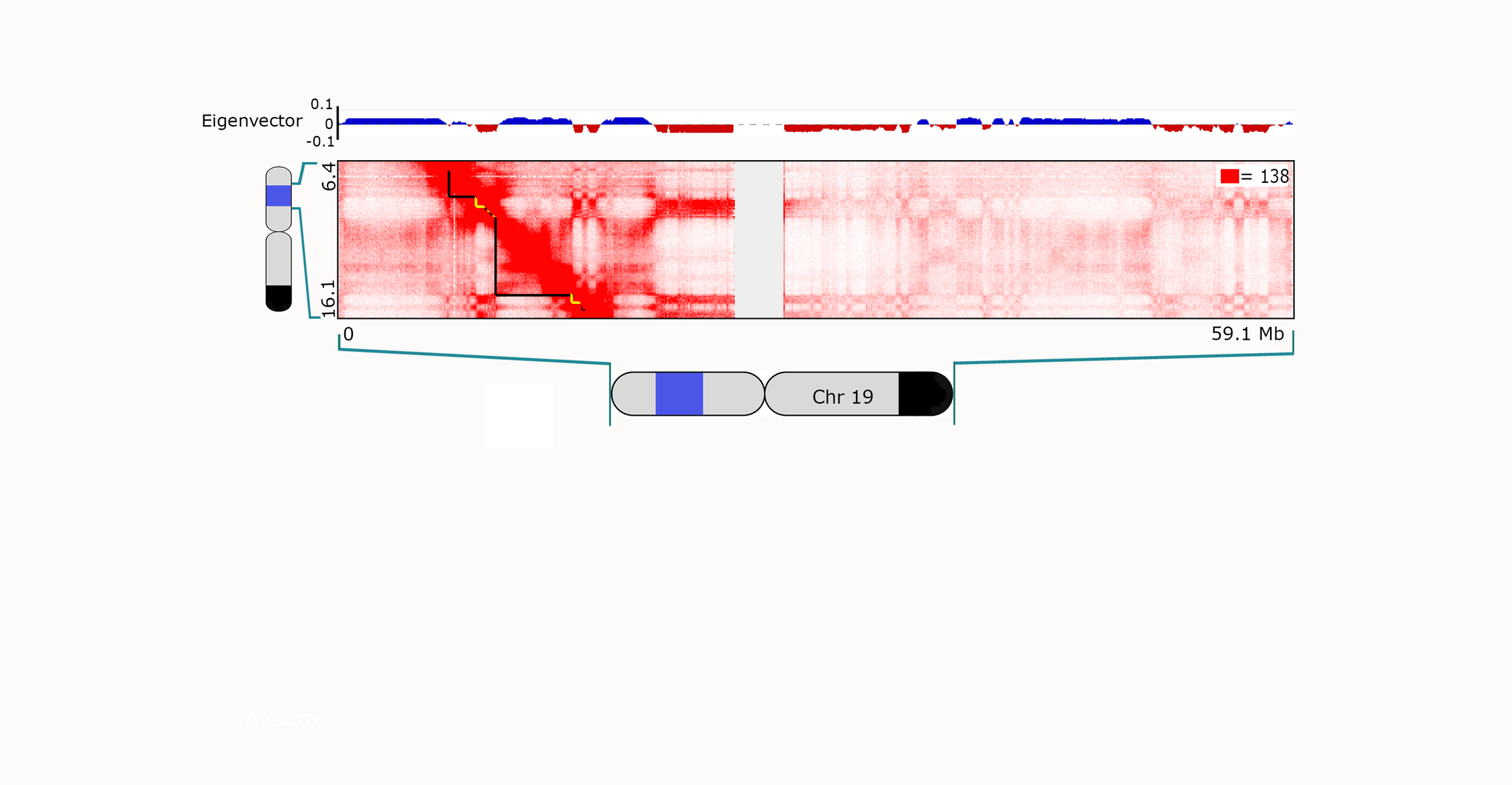

Supplement: S8 Fig — Map shows region including CS1-9, displayed at 100 kb resolution. (See Methods for data processing and normalization.) Contact count indicated by the color of each pixel, ranging from 0 (white) to 138 (red); manually curated intervals in compartments A and B are delineated below the diagonal in black and yellow, respectively, and reflect the positive values (blue) and negative values (red), respectively, of the compartment eigenvector at 100 kb resolution (above map). Cartoons of chromosome 19 show extent of the 8.16 Mb imaged region (blue) and block of SNVs shared between PGP1 and PGP95 (black; as in Fig 1B). (TIF) [file pgen.1007872.s019.tif]

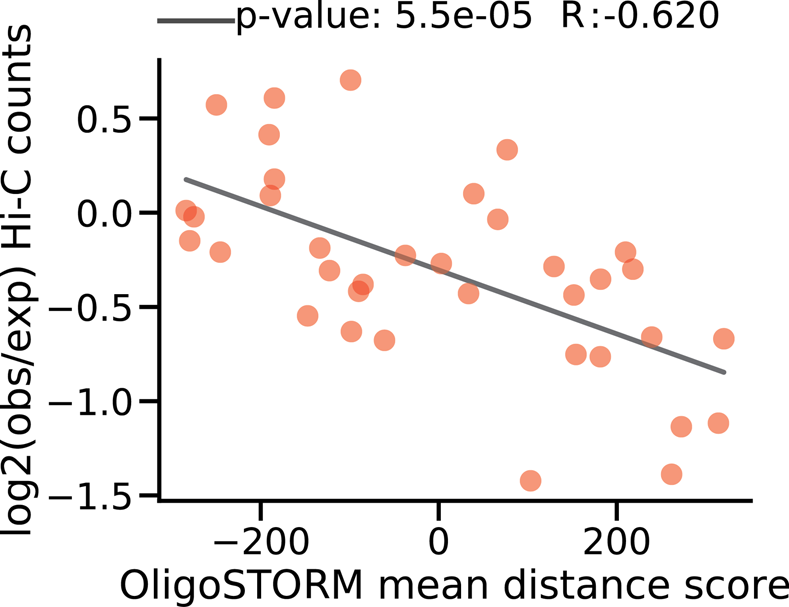

Supplement: S9 Fig — Correlation of Hi-C contact frequencies between chromosomal segments and corresponding mean distance scores. Hi-C contact frequency between chromosomal segments is expressed as log2(observed/expected) at 25 kb resolution. R = ~0.6 (p-value: 5.5e10-5). (TIF) [file pgen.1007872.s020.tif]

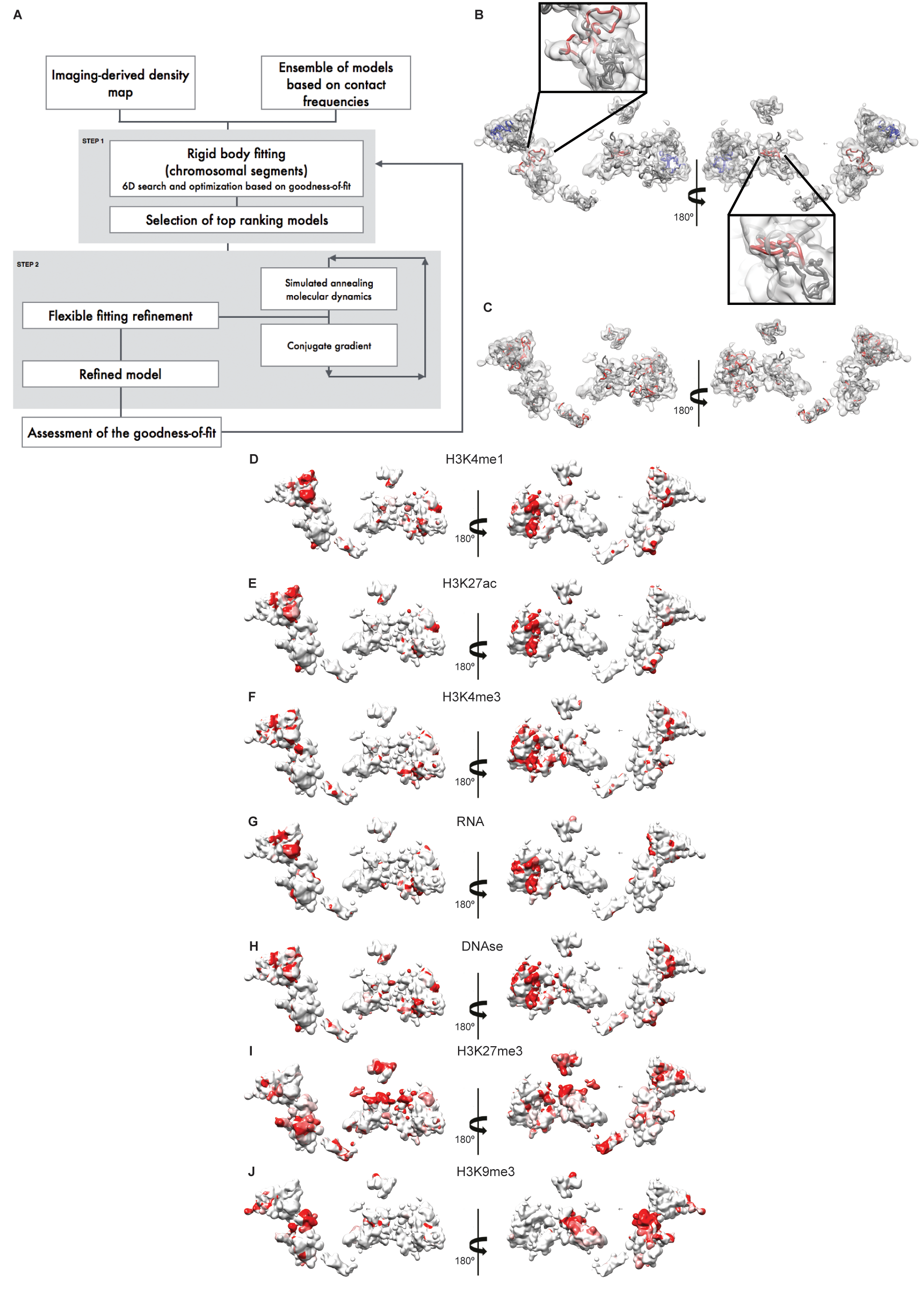

Supplement: S10 Fig — (A) IMGR protocol for fitting and refinement of 3D chromatin models. The protocol inputs are a density map derived from a single PGP1f nucleus imaged with OligoSTORM and an ensemble of 3D models derived from a PGP1f population-based in situ Hi-C contact map. The protocol entails rigid fitting by a random 6D search (step1), followed by flexible fitting refinement via simulated annealing rigid-body molecular dynamics and conjugate gradient minimization (step 2). After each step, the goodness-of-fit is assessed as described using different scores (Supporting information S1 Text). (B) Mapping of Znf clusters onto the 3D trace. The trace is colored in grey, and the two clusters are colored in blue and red. Insets are zoomed-in views of one of the Znf clusters, highlighting the differences in conformation of the cluster in the model of the two homologs. (C) Annotated disease-causing genes mapped into the 3D trace. The trace is colored in grey. Shown in red are genes for which the molecular basis for a disorder is known and a mutation has been found, as classified by OMIM Genes track in the UCSC Genome Browser [114]. (D-J) Mapping of genome features. Genomic features are presented as mean value per 10kb. The color scale for each genomic feature ranges from white (mean value) to red (mean + 2 standard deviation). The figures in this panel were created with Chimera [105]. (TIF) [file pgen.1007872.s021.tif]

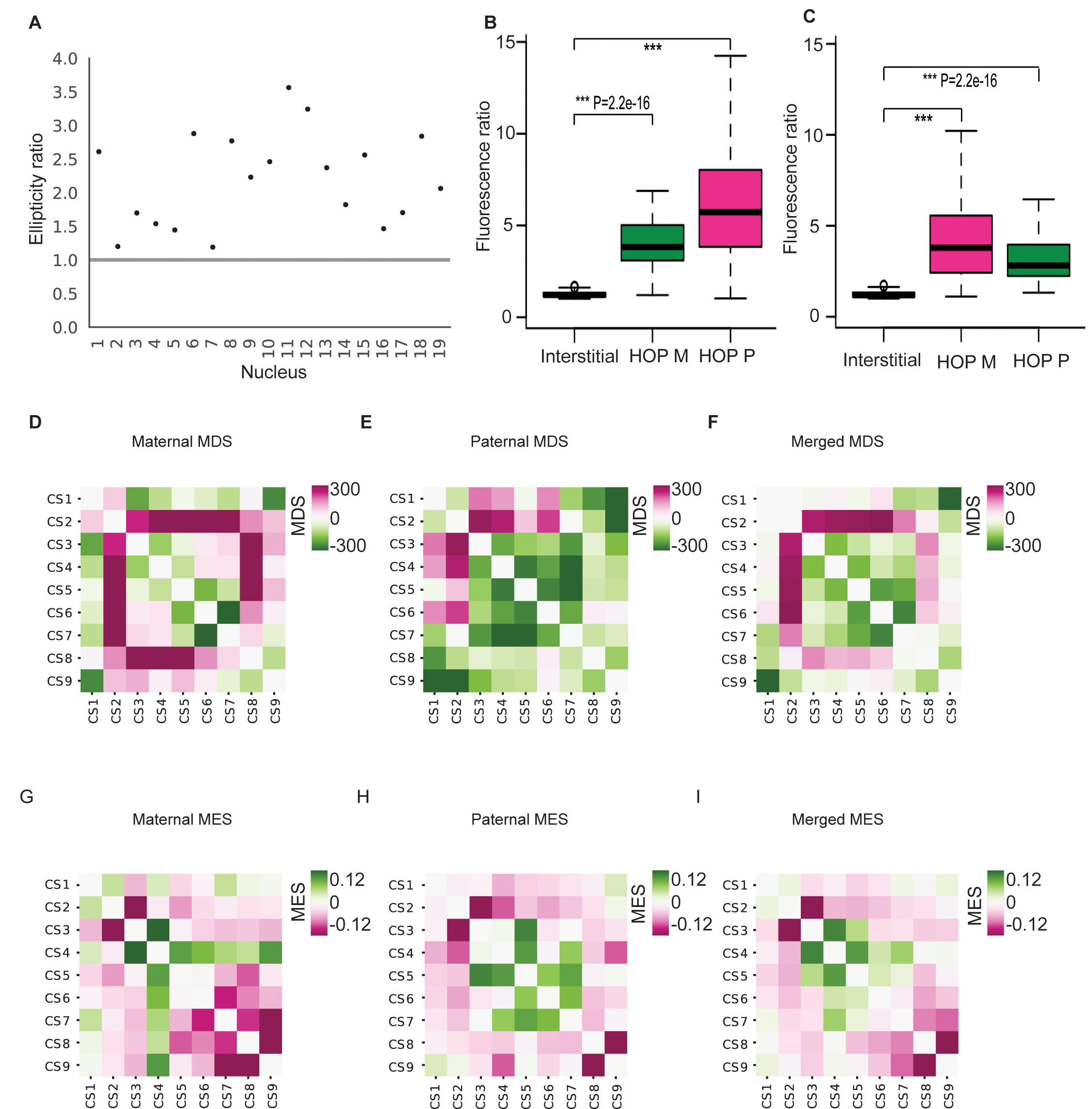

Supplement: S11 Fig — (A) Ellipticity ratios of the 19 nuclei analyzed (see S6 Table for values of ellipticity ratios). Grey line, ellipticity score (1.0) expected when the two largest principal axes are equal. (B) Ratios of signal intensity using interstitial, HOP-M (Atto488N), and HOP-P (Atto565N) probes. (C) Same as B, except HOP-M is now labeled with Atto565N, and HOP-P is labeled with Atto488N. (D-I) The MDS and MES matrices for six nuclei showing only (D, G) maternal homologs, (E, H) only paternal homologs, and (F, I) maternal and paternal homologs, combined. (TIF) [file pgen.1007872.s022.tif]

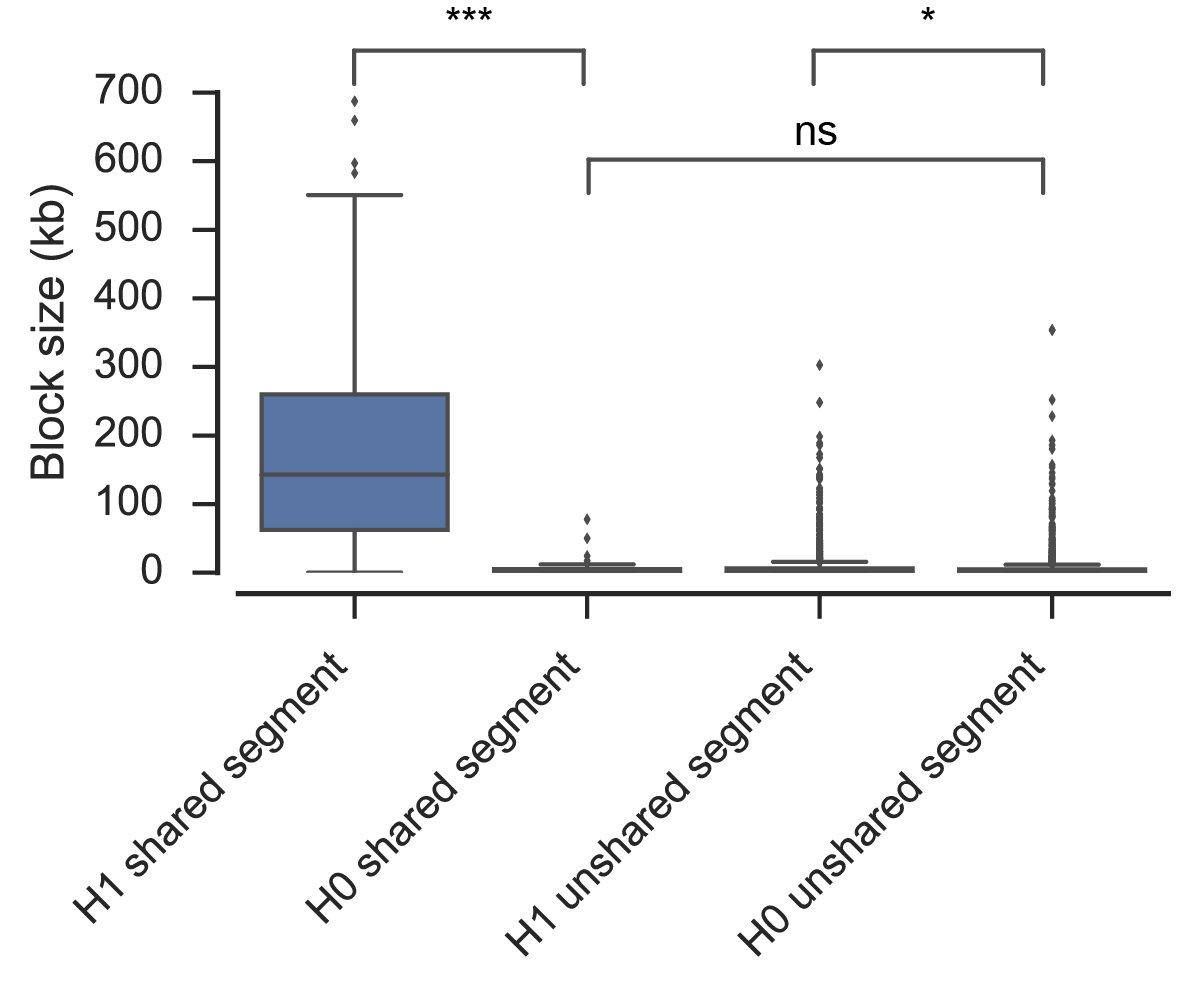

Supplement: S12 Fig — The longest blocks of SNVs on chromosome 19 of PGP1 that are shared with PGP95 correspond to the H1 haplotype, thus identifying homologs bearing long blocks of H1 SNVs as maternally derived (Mann-Whitney rank test, ns: non-significant, *: p < 5 x 10^−2^, ***: p < 10−3). (TIF) [file pgen.1007872.s023.tif]

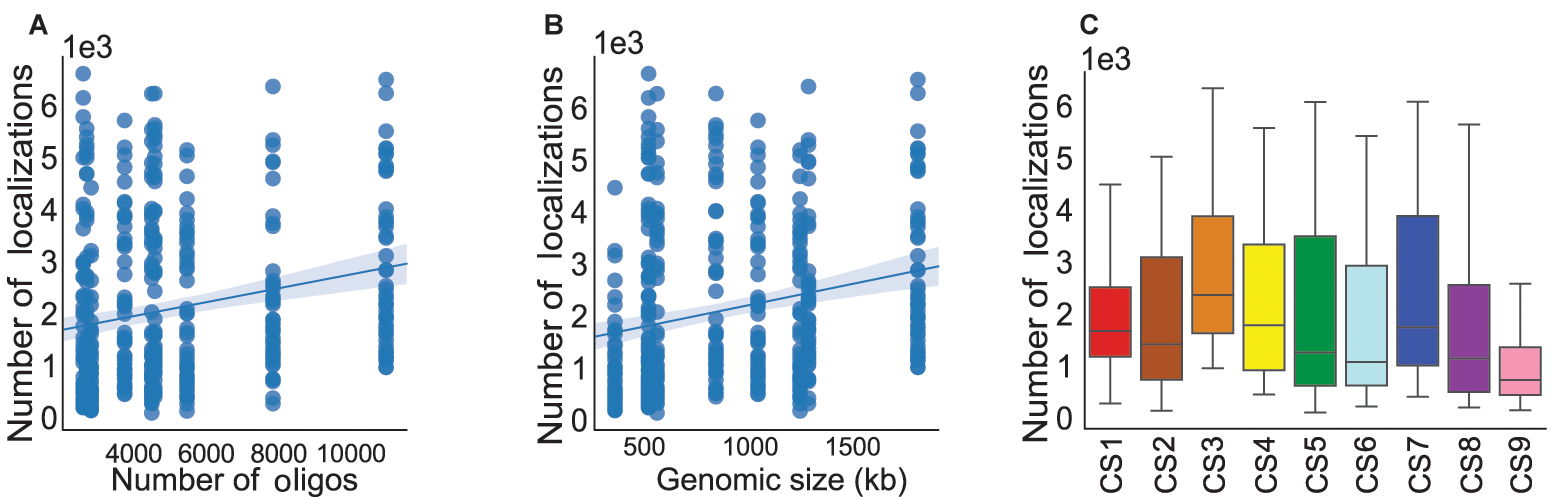

Supplement: S13 Fig — (A) Number of localizations versus number of oligos and (B) genomic distance for the 342 analyzed chromosomal segments (linear fit in blue). (C) Number of localizations per round of imaging. Color coding as in S7 Fig. (TIF) [file pgen.1007872.s024.tif]

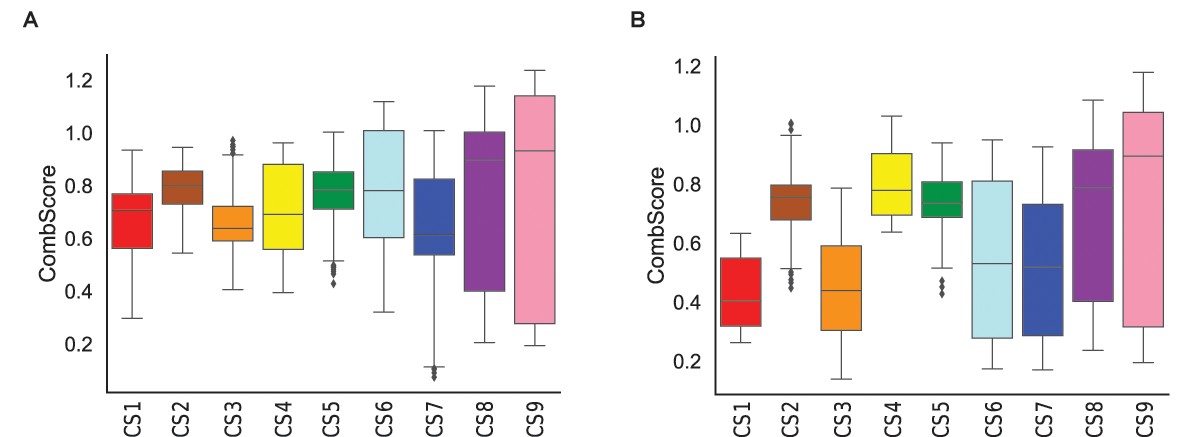

Supplement: S14 Fig — (A, B) Distribution of the Combined Score (CombScore) for each chromosomal segment of each of the two homologs traced. The chromosomal segments are color-coded as in Fig 2A and 2B. (TIF) [file pgen.1007872.s025.tif]

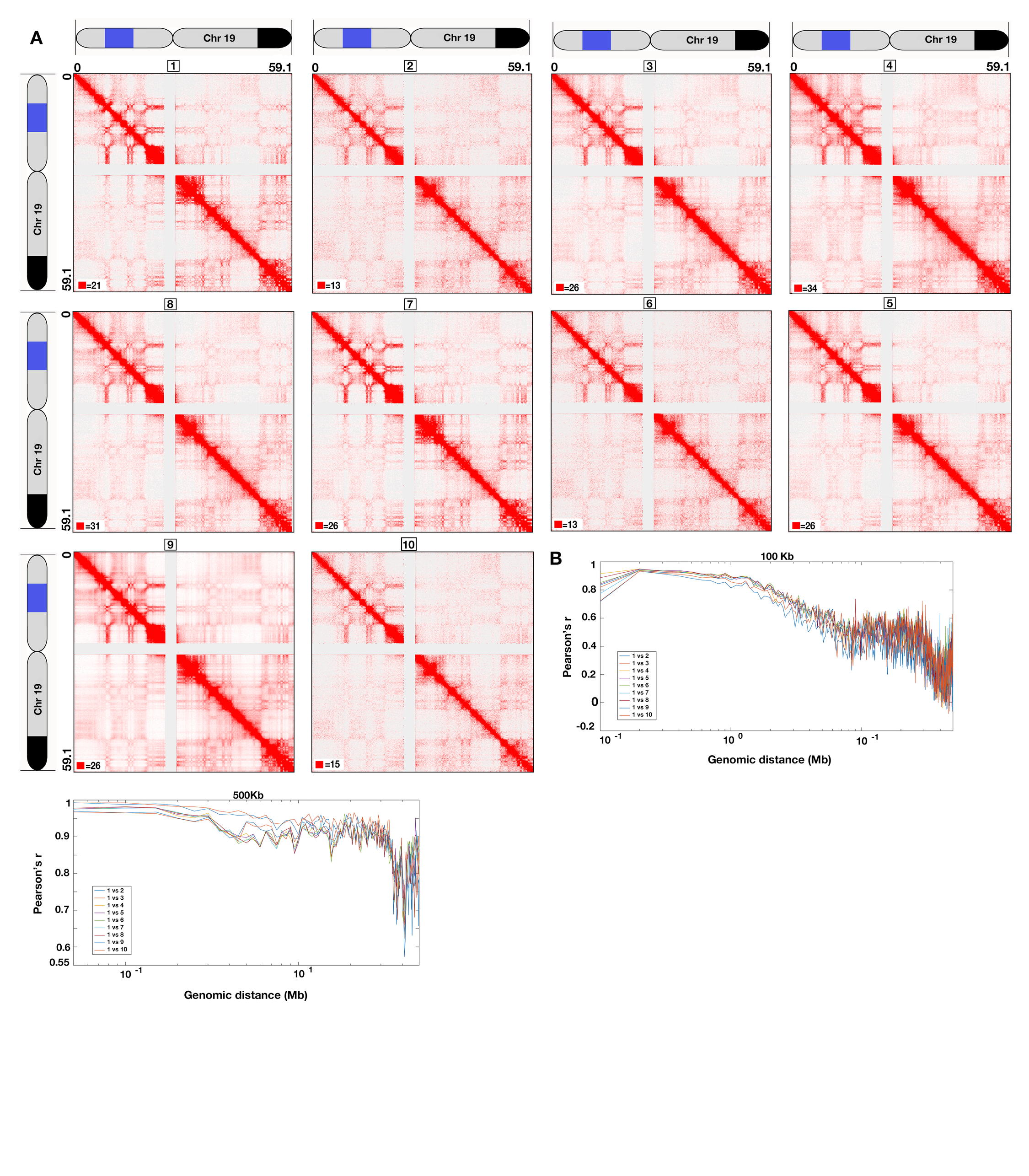

Supplement: S15 Fig — (A) The outcomes of ten Hi-C experiments conducted on PGP1f cells are extremely similar, as shown here and in S9 Table. The Hi-C maps represent the entirety of chromosome 19, displayed at 100 kb resolution. (See Methods for data processing and normalization.) Contact count indicated by the color of each pixel, ranging from 0 (white) to 34 (red). Cartoons of chromosome 19 show extent of the 8.16 Mb imaged region (blue) and block of SNVs shared between PGP1 and PGP95 (black; as in Fig 1B). (B) Correlation between in-situ Hi-C PGP1f libraries as a function of distance; Library 1 vs libraries 2–10 are shown at 100 and 500 kb resolutions. Hi-C replicates are highly correlated as demonstrated by the Pearson’s r value. At 500 kb resolution, r > 0.9 for distances of tens of megabases and 0.99 > r > 0.95 at a distance of 1 Mb for all replicates. At 100 kb resolution, r > 0.85 at a distance of 1 Mb for all replicates. Our results indicate that PGP1f libraries are highly similar. (TIF) [file pgen.1007872.s026.tif]
